# Potential Exposures to Australian Bat Lyssavirus Notified in Queensland, Australia, 2009−2014

**DOI:** 10.1371/journal.pntd.0005227

**Published:** 2016-12-29

**Authors:** Damin Si, John Marquess, Ellen Donnan, Bruce Harrower, Bradley McCall, Sonya Bennett, Stephen Lambert

**Affiliations:** 1 Communicable Diseases Branch, Queensland Health, Brisbane, Australia; 2 Forensic and Scientific Services, Queensland Health, Brisbane, Australia; 3 Metro South Public Health Unit, Metro South Hospital and Health Service, Queensland Health, Brisbane, Australia; 4 UQ Child Health Research Centre, School of Medicine, The University of Queensland, Brisbane, Australia; Wistar Institute, UNITED STATES

## Abstract

**Background:**

Australian bat lyssavirus (ABLV) belongs to the genus *Lyssavirus* which also includes classic rabies virus and the European lyssaviruses. To date, the only three known human ABLV cases, all fatal, have been reported from Queensland, Australia. ABLV is widely distributed in Australian bats, and any bite or scratch from an Australian bat is considered a potential exposure to ABLV.

**Methodology/Principal Findings:**

Potential exposure to ABLV has been a notifiable condition in Queensland since 2005. We analysed notification data for potential exposures occurring between 2009 and 2014. There were 1,515 potential exposures to ABLV notified in Queensland, with an average annual notification rate of 5.6 per 100,000 population per year. The majority of notified individuals (96%) were potentially exposed to ABLV via bats, with a small number of cases potentially exposed via two ABLV infected horses and an ABLV infected human. The most common routes of potential exposure were through bat scratches (47%) or bites (37%), with less common routes being mucous membrane/broken skin exposure to bat saliva/brain tissue (2.2%). Intentional handling of bats by the general public was the major cause of potential exposures (56% of notifications). Examples of these potential exposures included people attempting to rescue bats caught in barbed wire fences/fruit tree netting, or attempting to remove bats from a home. Following potential exposures, 1,399 cases (92%) were recorded as having appropriate post-exposure prophylaxis (PEP) as defined in national guidelines, with the remainder having documentation of refusal or incomplete PEP. Up to a quarter of notifications occurred after two days from the potential exposure, but with some delays being more than three weeks. Of 393 bats available for testing during the reporting period, 20 (5.1%) had ABLV detected, including four species of megabats (all flying foxes) and one species of microbats (yellow-bellied sheathtail bat).

**Conclusions/Significance:**

Public health strategies should address the strong motivation of some members of the public to help injured bats or bats in distress, by emphasising that their action may harm the bat and put themselves at risk of the fatal ABLV infection. Alternative messaging should include seeking advice from professional animal rescue groups, or in the event of human contact, public health units. Further efforts are required to ensure that when potential exposure occurs, timely reporting and appropriate post-exposure prophylaxis occur.

## Author Summary

Australian bat lyssavirus (ABLV), closely related to classic rabies virus, is widely distributed in bats in Australia. So far, the only three known human ABLV cases, all fatal, have been reported in Queensland, Australia. Any Australian bat-related injury (bite or scratch), or contact of bat saliva/neural tissue with eyes, mouth, nose, or broken skin of humans, is considered a potential exposure to ABLV. Use of timely, appropriate rabies vaccine and human rabies immunoglobulin is recommended to prevent fatal human ABLV disease. Based on notification data for potential exposures to ABLV in Queensland, we found that intentional handling of bats by the general public was the major cause of bat-related injuries; for example, people reported attempting to rescue bats caught in barbed wire fences or fruit tree netting, or attempting to remove bats from a home. This highlights a need to address the strong motivation of some members of the public to help injured bats or bats in distress and the lack of awareness of the risks of contact with or handling of bats, underscoring the importance of avoidance of bat handling by contacting vaccinated, experienced, and well-equipped professional animal rescue groups to deal with bats. There is a need to improve timeliness of notification to ensure immediate post-exposure management. Queensland Health bat testing results revealed the detection of ABLV in four common species of megabats (all flying foxes) and one species of microbats (yellow-bellied sheathtail bat), further reinforcing the message of avoiding all bat-related injuries. Once a potential exposure to ABLV occurs, immediate cleansing of wound (with soap and water for at least 5 minutes and application of povidone-iodine or alcohol) is required; and appropriate and timely rabies vaccine and human rabies immunoglobulin should be administered.

## Introduction

Australian bat lyssavirus (ABLV) is a member of the *Rhabdoviridae* virus family, genus *Lyssavirus*. There are 12 recognised species (genotypes) within the genus *Lyssavirus*, including classic rabies virus and other closely related lyssaviruses (e.g. European bat lyssaviruses) [1, 2]. Lyssaviruses are usually transmitted to humans via bites or scratches from an infected animal, which inoculate virus-laden animal saliva through human skin into muscle and subcutaneous tissues [3].

**Fig 1:**
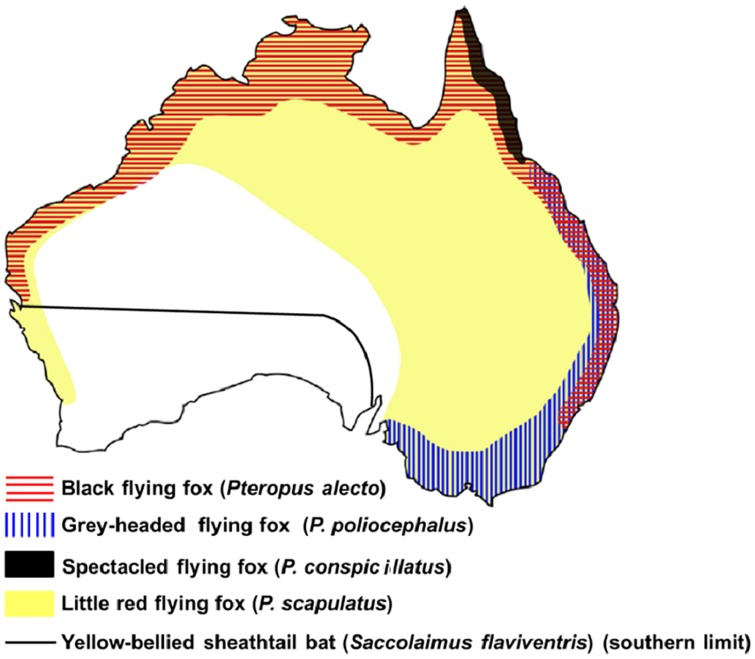
Distribution of ABLV host reservoir bat species in mainland Australia, reproduced from [5] under a CC BY license.

ABLV was first identified in Australia in 1996 from the brain of a black flying fox (*Pteropus alecto*) in New South Wales [4]. There has been virological evidence of ABLV infection in four common species of megachiroptera (megabat, including flying fox) in mainland Australia, and one species of microchiroptera (microbat) [1] [5]. These five ABLV reservoir bat species are widely distributed along the seaboard and inland Australia (Fig 1). Further serological evidence of ABLV infection has been reported in five of the six families of microbats in Australia [5]. Therefore, it is considered that all Australian bats have the potential to transmit ABLV.

To date, the only three known human cases of ABLV, all fatal, have occurred in Queensland, Australia. Cases occurred in 1996 [6], 1998 [7], and 2013 [8], with each case having a history of bat bite/s and/or scratch/es within Queensland.

In Australia, a bite or scratch by a bat, or mucous membrane/broken skin exposure to bat saliva or neural tissue, constitutes a potential exposure to ABLV. Under Queensland’s *Public Health Act 2005* [9], potential exposure to ABLV is a notifiable condition requiring immediate reporting by telephone call or facsimile to the local public health unit.

In this paper, we report on 1) notifications of potential exposures to ABLV in Queensland between 2009 and 2014; 2) the nature of and reasons for potential exposures; 3) provision of post-exposure prophylaxis; and 4) ABLV detection among bats involved in potential human exposure and available for testing.

## Methods

### Notification process and data collection

As a notifiable condition in Queensland, potential exposure to ABLV was defined as any bite or scratch from a bat in Australia, or mucous membrane (eyes, nose, or mouth) or broken skin contact with the saliva or neural tissue of an Australian bat [2]. Similar exposure to other mammals in Australia with confirmed ABLV infection was also considered as a potential exposure to ABLV. For the reporting period there were two horses [10] and one human [8] with confirmed ABLV infection reported in Queensland, Australia.

During the study period, doctors treating any person potentially exposed to ABLV were required to immediately notify a Queensland Health public health unit (PHU) of the potential exposure. Officers at the PHU interviewed the treating doctor and/or the person potentially exposed to determine the circumstances of the potential exposure, arranged for the testing of the bat if available, and assessed the need for post-exposure prophylaxis. A case report form [11] was used to collect data on personal details, clinical information, potential exposure details, place of potential exposure, rabies vaccination and human rabies immunoglobulin (HRIG) history, and management with rabies vaccination and HRIG following the potential exposure. Queensland-wide data on potential exposures to ABLV were recorded on the Notifiable Conditions Systems (NoCS). Data for this paper were extracted from NoCS on 30 June 2015 for potential exposures occurring from 1 January 2009 to 31 December 2014.

ABLV testing in bats involved in potential human exposure was conducted by Queensland Health Forensic and Scientific Services, using both immunofluorescent antibody (IFA) test for detection of lyssaviral antigen and polymerase chain reaction (PCR) for detection of ABLV RNA on brain tissue [12].

### Data classification

#### Circumstances of potential exposures

A description of the circumstances surrounding potential exposures was recorded as free text. These circumstances were categorised into six mutually exclusive and collectively exhaustive groups as follows:

1. **General public, intentional contact with a bat**:
  - Intentional rescue of a bat—persons intentionally recovering injured bats or releasing trapped bats, in circumstances such as bats trapped in fences, fruit tree netting, or attacked by dogs/cats;
  - Intentional removal of a bat—persons intentionally removing bats entering a home or found on the ground; or
  - Intentionally approached/touched/fed a bat—e.g. children interacting with or handling a bat when it has entered a home, people patting bats in a wildlife enclosure.
2. **General public, accidental contact with a bat** inside a house or in a bat’s habitat. For example, a confined space (e.g. a wardrobe) that contained a bat was opened; walked through bushland and stepped on a bat; cutting bananas and interrupted bats roosting in the banana plant.
3. **Volunteer bat carers and their families** exposed as a result of handling a bat in their care.
4. **Occupational contact with a bat**–people came into contact with a bat as part of their professional duties (e.g. veterinarians, bat handlers, bat researchers).
5. **Contact with two ABLV infected horses**–exposure to saliva or neural tissue of the ABLV infected horses across broken skin or mucous membranes.
6. **Contact with an ABLV infected human**–exposure to saliva or neural tissue of an ABLV infected person across broken skin or mucous membranes.

#### Post-Exposure Prophylaxis (PEP)

PEP requirement was assessed based on national guidelines [1, 2], as summarised below:

- Previously immunised persons
  - 2 doses of rabies vaccine. Human rabies immunoglobulin (HRIG) not required.
- Non-immune persons
  - 4 doses of rabies vaccine plus HRIG.
  - Prior to 2012, 5 doses of rabies vaccine plus HRIG were recommended as per Australian Immunisation Handbook (9th edition, 2008) [13].
- Previous immunity unknown
  - Treat as ‘non-immune’–see above.
- For immunocompromised persons, additional doses of vaccine may be required [14].
- Rabies vaccine and HRIG not required
  - A bat testing result was available within 48 hours of potential exposure and was ABLV negative. In the event that PEP already commenced, PEP may be discontinued following the negative ABLV result from bat testing.
  - Exposures to bats which had been dead for more than 4 hours.
- Periods of HRIG shortage—Australia experienced shortages of HRIG from time to time during the review period, and HRIG prioritisation measures could be implemented at the recommendation of Communicable Diseases Network Australia [2].

For each notified potential exposure to ABLV, PEP use was assessed as ‘consistent with national guidelines at the time’ or ‘variation from national guidelines’.

### Statistical analysis

Notification rates were calculated using Queensland estimated resident populations for 2009–2014. Confidence intervals (95%) for notification rates were calculated to facilitate comparison over time and across regions and population groups. A cumulative frequency curve was used to illustrate the time lag between potential exposure dates and notification dates to public health units. Confidence intervals (95%) for the median time lags, calculated using 200 bootstrap samples, were adopted to facilitate comparison between groups. All data analyses were performed using Stata (version 14.1, College Station, TX, USA).

### Ethics review

Surveillance and management of notifiable conditions including potential exposures to ABLV is a routine practice regulated under Queensland’s *Public Health Act 2005* [9]. As a quality improvement audit, an exemption from ethics review was granted by the Forensic and Scientific Services Human Ethics Committee, Queensland Health (reference HEC16-04). No individual level patient-identifying data are presented in this paper.

## Results

### Notifications of potential exposures to ABLV, 2009–2014

There were 1,515 potential exposures to ABLV notified in Queensland residents over the period 2009–2014 (Table 1), with 1,500 occurring in Queensland and 15 occurring in other Australian states/territories. This gives an average annual notification rate of 5.6 potential exposures per 100,000 population per year. The highest notification rate was reported in 2013 (10.9 per 100,000 per year).

**Table 1:** Notifications of potential exposures to ABLV in Queensland, 2009–2014.

Year	Number of potential exposures	Notification rate* (95% confidence interval)
2009	113	2.5 (2.2–3.1)
2010	234	5.3 (4.7–6.0)
2011	155	3.5 (2.9–4.1)
2012	169	3.7 (3.2–4.3)
2013	508	10.9 (10.0–11.9)
2014	336	7.1 (6.4–7.9)
**2009–2014**	**1,515**	**5.6 (5.3–5.9)**

* Per 100,000 population per year

**Fig 2:**
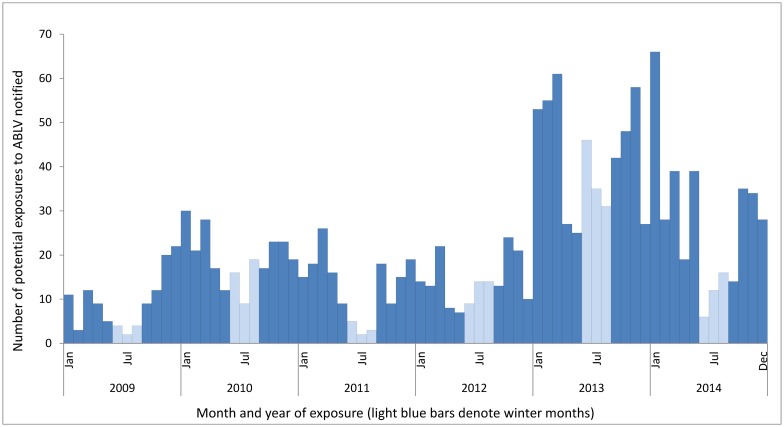
Potential exposures to ABLV notified in Queensland by month of potential exposure, 2009–2014.

Potential exposures occurred at all times of the year, with relatively fewer potential exposures in the winter months (June–August, 16% of total notifications) (Fig 2).

Time lags between potential exposure dates and notification dates to PHUs are shown in S1 Fig. Notifications to PHUs were made on the same day for 41% of potential exposures; 76% were notified within 2 days following potential exposure; 86% were notified within 7 days following potential exposure; and 93% were notified by 21 days. The median time from potential exposure to notification was 1 day. The longest period from potential exposure to notification was 3 years and 8 months (1,344 days). There was no statistically significant difference in median times from potential exposure to notification by gender, age group (under 40 year olds versus 40 years or older), and occupational group (those with occupational contact with bats versus the general public). Time lag patterns were similar for notifications in 2013 and those in other years (S2 Fig).

### Demographic details of people potentially exposed

The median age of people notified as having a potential exposure to ABLV was 42 years. The highest number of notifications occurs in the 40–49 year age group and the highest notification rate was in the 50–69 year age group (Table 2). Males had generally higher notification counts than females, but notification rates were not statistically different for the two groups.

**Table 2:** Notifications of potential exposures to ABLV by sex and age group, 2009–2014, Queensland.

Age group (years)	Male		Female		Total	
	Number of potential exposures	Notification rate* (95% CI)	Number of potential exposures	Notification rate* (95% CI)	Number of potential exposures	Notification rate* (95% CI)
00–09	57	3.0 (2.3–3.9)	52	2.9 (2.2–3.9)	109	3.0 (2.5–3.6)
10–19	83	4.5 (3.6–5.6)	55	3.1 (2.4–4.1)	138	3.8 (3.2–4.5)
20–29	114	5.7 (4.7–6.9)	113	5.8 (4.8–7.0)	227	5.8 (5.1–6.6)
30–39	118	6.3 (5.2–7.6)	116	6.2 (5.1–7.8)	234	6.2 (5.5–7.1)
40–49^#^	124	6.6 (5.5–7.9)	136	7.1 (5.9–8.4)	260	6.9 (6.0–7.7)
50–59	138	8.2 (6.9–9.7)	113	6.6 (5.4–7.9)	251	7.4 (6.5–8.4)
60–69	95	7.1 (5.8–8.7)	103	7.8 (6.4–9.5)	198	7.5 (6.5–8.6)
70–79	51	7.0 (5.2–9.2)	30	3.9 (2.7–5.6)	81	5.4 (4.3–6.8)
80+	15	4.2 (2.4–6.9)	1	0.2 (0.05–1.0)	16	1.8 (1.0–2.9)
**Total**	**795**	**5.9 (5.5–6.3)**	**719**	**5.3 (4.9–5.7)**	**1,514**	**5.6 (5.3–5.9)**

* Average annual notification rate per 100,000 population per year.

^#^ One notified case with unknown sex in this age group.

**Fig 3:**
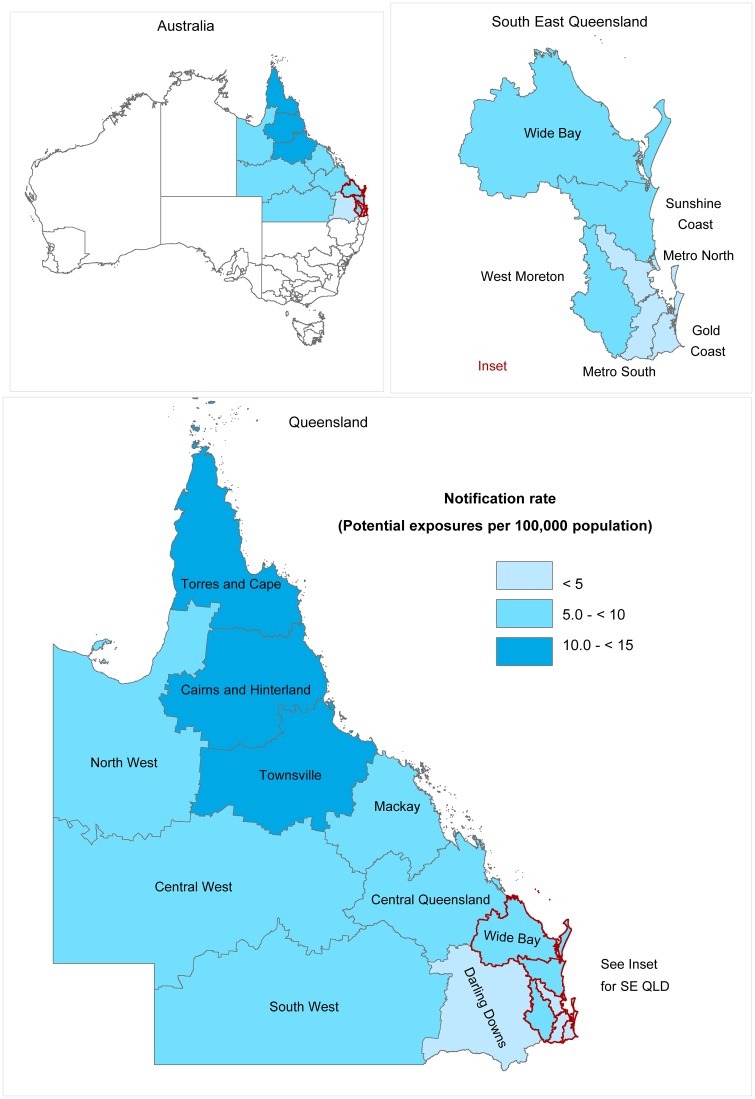
Average annual notification rates of potential exposures to ABLV by Hospital and Health Service of residence in Queensland, 2009–2014.

There was variation in average annual notification rates across geographical areas of residence (Fig 3). Notification rates ranged from 3.7 per 100,000 per year in Metro South to 12.8 per 100,000 per year in Cairns and Hinterland (S1 Table). Rates were generally higher in north Queensland (the tropical area) than in south Queensland (the temperate area).

### Nature of potential exposures to ABLV

#### Animals

Of 1,515 cases of potential exposures to ABLV, 1,461 (96.4%) were exposed via contact with bats, 8 (0.5%) via contact with two ABLV infected horses, 20 (1.3%) via contact with an ABLV infected human, and 26 (1.7%) via contact with animals thought to be bats. Of these 1,461 cases of potential exposures to ABLV via contact with bats, 1,001 reported contact with megabats (including 985 flying foxes), 254 contact with microbats, and 206 contact with unspecified bats.

#### Routes of potential exposure

The most common potential exposures were through bat scratches (709, 46.8%) and bites (565, 37.3%), with less common routes being mucous membrane/broken skin exposure to bat saliva/brain tissue (34, 2.2%), mucous membrane/broken skin exposure to horse/human saliva or brain tissue (28, 1.8%), and other close contact with bats, including situations during which infectious contact could not be excluded (179, 11.8%).

#### Circumstances of potential exposure

The most common circumstance of potential exposure was intentional contact with a bat by the general public (56.3%), followed by accidental contact with a bat by the general public (32.7%), occupational contact with bats (3.5%), and contact with bats by volunteer bat carers and their families (1.2%). Contact with two ABLV infected horses or an ABLV infected human occurred in 1.8% of notifications (Table 3).

**Table 3:** Circumstances of potential exposures to ABLV in Queensland, 2009–2014.

Circumstance of potential exposures	Number	%	Example of common activities (frequency)
General public, intentional contact with a bat	853	56.3	
Intentional rescue of a bat	513		Rescue of a bat caught in barbed wire fences (124)
			Rescue of a bat caught in fences (unspecified) (93)
			Rescue of a bat caught in fruit tree netting (104)
			Rescue of a bat attacked by dogs/cats (62)
			Other (130)
Intentional removal of a bat	192		Intentional removal of a bat from a room (72)
			Removal of a bat from ground, yard, or road (44)
			Other (76)
Intentionally approached/touched/fed a bat	148		-
General public, accidental contact with a bat	496	32.7	Accidental contact with a bat at home (142)
			During walk/jog/run (89)
			During bicycle riding (23)
			A bat which flew into open window of a car (16)
			Other (226)
Volunteer bat carers and their families, contact with bats	18	1.2	-
Occupational contact with bats	53	3.5	Staff of animal clinics (18)
			Veterinarians (8)
			Bat collectors/handlers (8)
			Bat researchers (6)
			Euthanasia of bats (5)
			Workers at wildlife sanctuary (2)
			Ecologist (1)
			Council worker (1)
			Other (4)
Contact with two ABLV infected horses [10]	8	0.5	-
Contact with an ABLV infected human [8]	20	1.3	-
Not reported	67	4.4	-
**Total**	**1,515**	**100.0**	

The most common activities for intentional contact with a bat by the general public included: the rescue of a bat caught in a fence (mostly described as barbed wire fences), in fruit tree netting, or following attack by dogs/cats; intentional removal of a bat from a room; and contact when a bat was touched or fed.

Accidental contact with a bat by the general public most commonly occurred when people encountered bats inside their houses, or during outdoor exercise.

Occupational contact with bats was reported in 53 cases, including staff of animal clinics (18), veterinarians (8), bat collectors/handlers (8), and bat researchers (6).

### Post-exposure prophylaxis

Of 1,515 notified cases of potential exposures to ABLV, 1,399 (92.3%) received PEP consistent with national guidelines at the time of potential exposure; the remaining 116 (7.7%) had PEP use varying from national guidelines (Table 4).

**Table 4:** Use of post-exposure prophylaxis (PEP) among notified cases of potential exposures to ABLV in Queensland, 2009–2014.

PEP use	Number	%
**Consistent with national guidelines at the time**	**1,399**	**92.3**
Appropriate doses of rabies vaccine and HRIG	1,151	
PEP not required/discontinued, bats tested negative for ABLV	244	
PEP not required, exposure to bats which had been dead for more than 4 hours	4	
**Variation from national guidelines**	**116**	**7.7**
Four or five doses of rabies vaccine per guidelines, but no documentation of HRIG use	95	
Patient refusal of recommended vaccine/HRIG	3	
Partial vaccine receipt only documented	6	
HRIG receipt only documented	5	
No PEP data documented	7	
**Total**	**1,515**	**100.0**

### ABLV testing in bats

Over the period 2009–2014, 393 bats involved in ABLV potential exposure incidents were available for testing (Table 5). Most of these bats were retrieved in circumstances where people intentionally rescued sick/injured bats or had occupational exposure to bats. Of those bats, 20 had ABLV detected, with an overall ABLV detection rate of 5.1% in bats tested. In addition, fourteen bats tested equivocal for ABLV by Immunofluorescent Antibody (IFA) testing but negative for ABLV by PCR method.

**Table 5:** Detection of ABLV in bats available for testing, 2009–2014.

Year	Number of bats tested	ABLV detected*		Equivocal ABLV^#^	
		Number	%	Number	%
2009	47	4	8.5	2	4.3
2010	83	4	4.8	0	0.0
2011	48	3	6.3	1	2.1
2012	58	1	1.7	0	0.0
2013	97	2	2.1	4	4.1
2014	60	6	10.0	7	11.7
**2009–2014**	**393**	**20**	**5.1**	**14**	**3.6**

* ABLV detected in both Immunofluorescent Antibody (IFA) and Polymerase Chain Reaction (PCR) tests.

^#^ IFA tested equivocal for ABLV, but ABLV was not detected by PCR.

Details on bat species and ABLV testing results are shown in Table 6. Bats classified as *Pteropus* genus (megabats) accounted for 80% (314/393) of bats tested, and 19 of 20 (95%) bats testing positive for ABLV belonged to this genus. The ABLV detection rate was 6.0% (19/316) among megabats tested and 1.6% (1/64) among microbats tested. For individual bat species, the ABLV detection rate was 14.9% among *Pteropus scapulatus*, followed by *Pteropus conspicillatus* (7.7%), *Pteropus poliocephalus* (7.7%), and *Pteropus alecto* (2.6%). Of 3 *Saccolaimus flaviventris* microbats tested, one (33.3%) was ABLV positive.

**Table 6:** Bat species and ABLV testing results, 2009–2014.

Bat species	Number of bats tested	ABLV detected*		Equivocal ABLV^#^	
		Number	%	Number	%
**Megachiroptera (megabat)**					
*Pteropus* species					
*Pteropus alecto*	195	5	2.6	5	2.6
*Pteropus conspicillatus*	13	1	7.7	0	0.0
*Pteropus poliocephalus*	39	3	7.7	0	0.0
*Pteropus scapulatus*	67	10	14.9	0	0.0
*Syconycteris australis*	2	0	0.0	0	0.0
**Microchiroptera (microbat)**					
*Chaerephon jobensis*	1	0	0.0	0	0.0
*Chalinolobus gouldii*	1	0	0.0	0	0.0
*Miniopterus* species					
*Miniopterus australis*	3	0	0.0	1	33.3
*Miniopterus schreiber*	1	0	0.0	0	0.0
*Miniopterus* species (unspecified)	1	0	0.0	0	0.0
*Mormopterus* species					
*Mormopterus beccarii*	11	0	0.0	3	27.3
*Mormopterus norfolken*	2	0	0.0	0	0.0
*Mormopterus* species (unspecified)	8	0	0.0	1	12.5
*Nyctinomus australis*	3	0	0.0	0	0.0
*Nyctophilus* species					
*Nyctophilus bifax*	5	0	0.0	0	0.0
*Nyctophilus gouldii*	5	0	0.0	1	20.0
*Nyctophilus* species (unspecified)	2	0	0.0	0	0.0
*Saccolaimus flaviventris*	3	1	33.3	0	0.0
*Scoteanax rueppellii*	3	0	0.0	0	0.0
*Scotorepens* species					
*Scotorepens greyii*	1	0	0.0	0	0.0
*Scotorepens orion*	8	0	0.0	0	0.0
*Scotorepens* species (unspecified)	3	0	0.0	1	33.3
*Vespadelus pumilus*	1	0	0.0	0	0.0
*Vespertilionidae* family	2	0	0.0	0	0.0
**Bat—unspecified**	13	0	0.0	2	15.4
**Total**	**393**	**20**	**5.1**	**14**	**3.6**

* ABLV detected in both Immunofluorescent Antibody (IFA) and Polymerase Chain Reaction (PCR) tests.

^#^ IFA tested equivocal for ABLV, but ABLV was not detected by PCR.

## Discussion

There are two key findings that emerge from our review of six years of potential human exposures to ABLV in Queensland. Firstly, potential exposure most commonly occurred through intentional contact with bats. Secondly, up to a quarter of notifications occurred after two days from the potential exposure, but with some delays being more than three weeks. Both of these findings highlight avoidable risks in terms of potential exposure and preventing disease progression, and should continue to be part of the key messages in future health promotion and public education campaigns on this topic.

Timely and appropriate PEP remains the only effective measure in preventing human ABLV infection following scratches/bites by bats or mucosal contact with bats’ saliva/neural tissue [2, 15]. The incubation period of classic rabies is usually 3–8 weeks (but can be highly variable). The incubation period for the three known human ABLV cases has ranged from 4 weeks to 27 months [6–8]. Immediate management of any potential exposure is recommended [2], making any delay in notification potentially very serious. It is important that individuals potentially exposed to ABLV seek medical care immediately to receive timely post-exposure prophylaxis. Our data show that when a potential exposure was notified, there was a high level of appropriate PEP administration, with 92% of notified cases with PEP use consistent with national guidelines. This included approximately 16% of notified cases who did not require PEP or had their PEP discontinued due to confirmation of negative ABLV testing results in bats. The remainder all had either refusal of PEP or partial PEP documented. Where partial PEP was documented, the majority had appropriate doses of vaccine but with no documentation of HRIG. Importantly, retrieval of bats for laboratory testing should be performed by trained, vaccinated, and well equipped professional bat handlers, to avoid further exposure from bat bites/scratches.

Statewide notification data demonstrate that 56% of potential exposures to ABLV were from members of the public who intentionally handled bats, most commonly in circumstances such as rescue of trapped bats from fences or fruit tree netting, or removal of bats from a home. Fewer than 5% of reported potential exposures were in professional groups (e.g. veterinarians, bat handlers) and volunteer bat carers. Previous data from the Brisbane South PHU [16–18] showed that professional bat handlers and volunteer bat carers accounted for 50% of potential exposure cases in 1996–1999 (the first two fatal human ABLV cases were reported in 1996 [6] and 1998 [7]), but the proportion reduced to around 20% in 2000–2008. In contrast, the proportion of potential exposures reported by the general public who intentionally handled bats increased from approximately 40% to 60% over the same period [18]. Public health information campaigns to raise awareness of ABLV risks targeted professional and volunteer groups handling bats, which may have partially contributed to the substantial decline in the proportion of potential exposures from these groups [16]. There may also be potential for under-reporting among volunteer bat carer groups due to requirement of bat euthanasia for testing [17]. However, a sustained high proportion of potential exposures from the general public who continue to handle bats intentionally highlights a continuing need to address the strong motivation of some members of the public to help bats in distress.

Recent surveys of community members in Queensland [19] and New South Wales [20] shed light on perception of bat risk and intention to handle bats from the general population. In 2014 Young *et al* [19] reported a survey of residents in South East Queensland and found that 20% (140/700) of participants considered bats as a high risk to human health, 31% considered a moderate risk, and 42% considered a low risk. Approximately 25–30% of participants from the survey indicated they would handle a bat trapped in wire mesh fencing or a net over a fruit tree, and 56% would handle a bat coming into their houses. Similarly, a population-based survey in New South Wales [20] reported that a quarter of respondents would handle an injured or trapped bat. Key reasons for people reporting a willingness to handle bats included [19]: 1) protection of their families, pets, and themselves from bats, in situations such as a dead bat in the yard or a live bat in the house; 2) protection of injured or trapped bats; and 3) perception of minimal or no risk from injury if implements, gloves, or towels were used to handle bats.

The largest number of potential exposures to ABLV (508 individuals) was notified in 2013. This was probably attributable to increased community awareness of ABLV in the context of bat exposures following the reporting (both through Queensland Health and the media) of a human death from ABLV in February 2013 [8], and of the first confirmed ABLV infections in two horses in May 2013 [10].

Given the considerable proportion of people (approximately a quarter) in the community who reported an intention to handle injured or trapped bats [20], public health interventions should adopt multiple strategies, including: 1) communicating the risk messages to the general public that they may harm the bat and put themselves at risk of contracting the fatal ABLV infection if they attempt to rescue/handle the animal [21], because an untrained person is highly likely to be injured when handling a bat, and the bat involved in human injury will be euthanised for ABLV testing. 2) advising the public to take an alternative action when encountering injured/trapped bats; for example, contacting animal rescue groups (e.g. RSPCA) [22] to handle bats, as these professionals are vaccinated, experienced and well equipped to deal with bats; 3) collaboration with industry for better design of fences and fruit tree netting to minimise bats being trapped; and 4) education on the need for immediate cleansing of wound (with soap and water for at least 5 minutes and application of povidone-iodine or alcohol) when indicated, and to immediately seek medical attention following bat scratches/bites or mucosal membrane/broken skin contact with bat saliva/neural tissue.

Our bat testing data confirmed that ABLV was detected in four species of megabats (all flying foxes) and one species of microbats over the reporting period: *Pteropus scapulatus* (common name—little red flying fox), *Pteropus alecto* (black flying fox), *Pteropus conspicillatus* (spectacled flying fox), *Pteropus poliocephalus* (grey-headed flying fox), and *Saccolaimus flaviventris* (yellow-bellied sheathtail bat). Currently flying fox camp locations and populations have been monitored in Australia through the National Flying Fox Monitoring Programme [23]. There are two distinct variants of ABLV: ABLVp found in *Pteropus* bats and ABLVs found in *Saccolaimus* bats [5].

Based on our data, 5.1% of bats tested were ABLV infected (6.0% for megabats and 1.6% for microbats). Recent data from Wildlife Health Australia showed the prevalence of ABLV in bats submitted for testing was 6.7% [24]. For individual species, the little red flying fox had relatively high prevalence of ABLV infection (14.9% in our data, 17.5% reported by Field [25], and 16.9% by Barrett [26]). It was noted that of 3 yellow-bellied sheathtail microbats tested, one (33%) was ABLV positive. A previous study found 5 of 7 (71.4%) yellow-bellied sheathtail bats tested ABLV positive [26], an indication of extremely high prevalence of ABLV among such bat species. The ABLV infection rate in sick, injured, or orphaned bats with central nervous symptoms could be as high as 21% [26]. Sick or injured bats are more likely to be involved in human-bat interactions [20]. These findings further reinforce the message of avoiding any bat handling by the general public.

Eight people were exposed to two ABLV infected horses which were first confirmed in 2013 [10]. Spillover of ABLV from natural reservoir bats to horses raises the question of whether ABLV can transmit to other animals, such as dogs. Contact between domestic dogs and ABLV infected bats has been reported previously [27], and our data indicate occasional pet animal-bat interactions (dogs/cats attacking bats on 62 occasions). Molecular studies on lyssavirus host cell entry mechanisms have found that ABLV host cell receptor is broadly conserved among mammals [28], indicating these animals are likely to be susceptible to ABLV infection. Future research is needed to better understand the potential transmission of ABLV from bats to domestic animals and implications for public health.

Some caution is needed when interpreting our notification data. Firstly, the number of potential exposures to ABLV in Queensland was likely to be under-reported. The trivial nature of the injury, coupled with lack of awareness of serious risk associated with bat-related injury, and subsequent failure to seek medical attention by some individuals potentially exposed to ABLV are likely to contribute to under-reporting. It is important that any person with bat-related injury sees a doctor immediately—no matter how small the injury is [15]. Secondly, applying the case definition on what constitutes a potential exposure to ABLV in public health practice can be challenging in some situations and careful consideration of the use of HRIG as a scarce resource is required. For instance, providing PEP to a person who: is scratched by a flying creature at night when that creature has not been identified as a bat; or awakens to a bat in the bedroom but is not aware of any exposure. Consideration for PEP on a case by case basis is warranted in public health practice to prevent rare but fatal disease. Thirdly, there was insufficient documentation of reasons to explain why PEP use in some individuals varied from guidelines. Our data showed 95 people had appropriate doses of rabies vaccine, but had no information on why HRIG was not documented. HRIG rationing measures applied during periods of HRIG shortage might contribute to restriction of HRIG use in some of these cases, however there is a need to clearly document the role of these restrictions in the PEP recommended.

While human disease from ABLV is uncommon, historically, identified clinical disease has been universally fatal. Further efforts are required to minimise potentially infectious contact between humans and bats, and to ensure that timely notification and appropriate post-exposure prophylaxis is administered following potential exposures to ABLV.

## Supporting Information

S1 Fig Time lags between potential exposure dates and notification dates for notified potential exposures to ABLV, 2009–2014. (TIF)

S2 Fig Time lags between potential exposure dates and notification dates for notified potential exposures to ABLV, 2013 vs. other five years combined (2009–2012 plus 2014). (TIF)

S1 Table Notification rate for potential exposures to ABLV by Hospital and Health Service of residence, 2009–2014, Queensland. (DOCX)
