# Supplementary figures and images for: Potential Exposures to Australian Bat Lyssavirus Notified in Queensland, Australia, 2009−2014
Source: PLoS Negl Trop Dis. 2016 Dec 29;10(12):e0005227. doi: 10.1371/journal.pntd.0005227 (PMC5199083; doi:10.1371/journal.pntd.0005227)

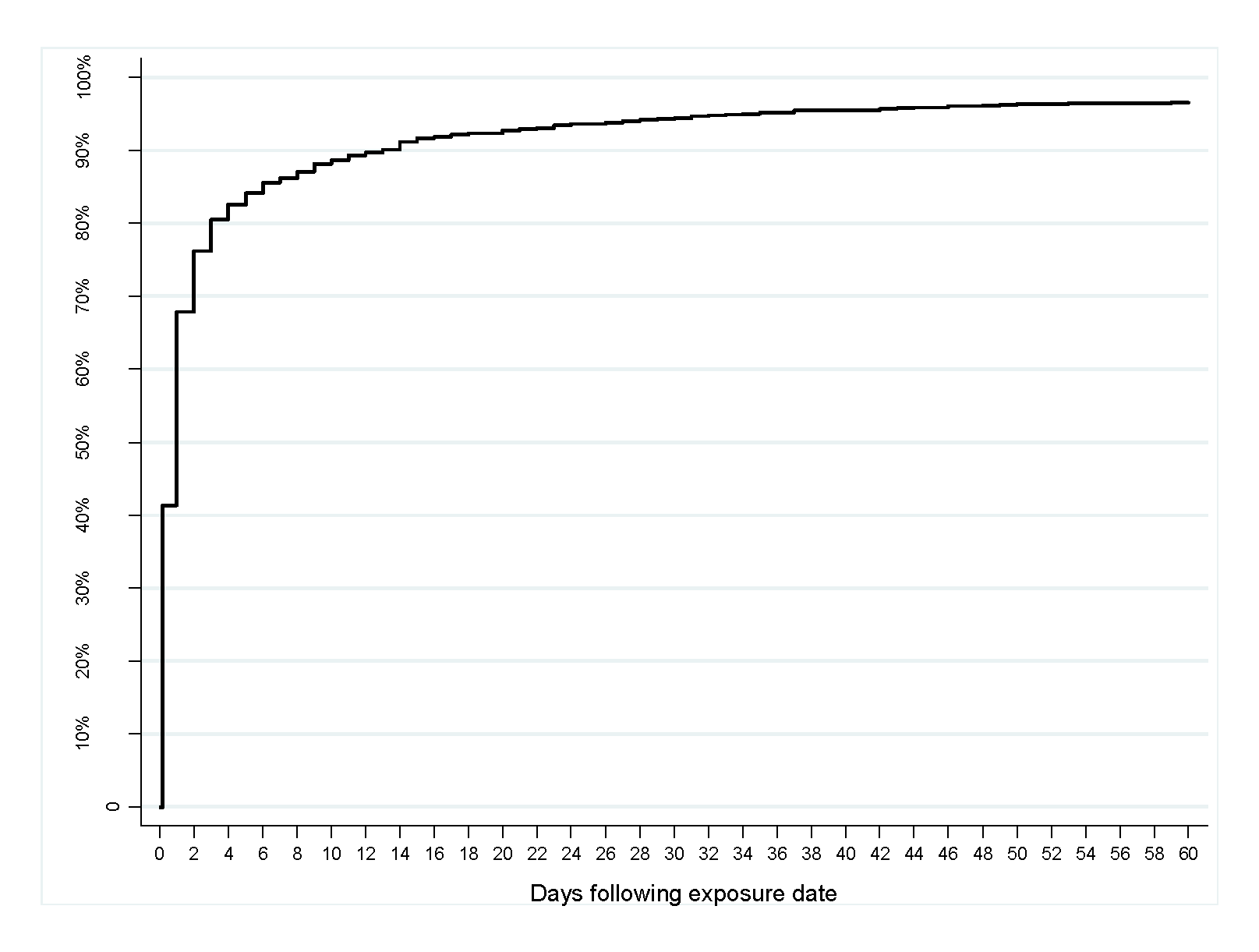

Supplement: S1 Fig — (TIF) [file pntd.0005227.s001.tif]

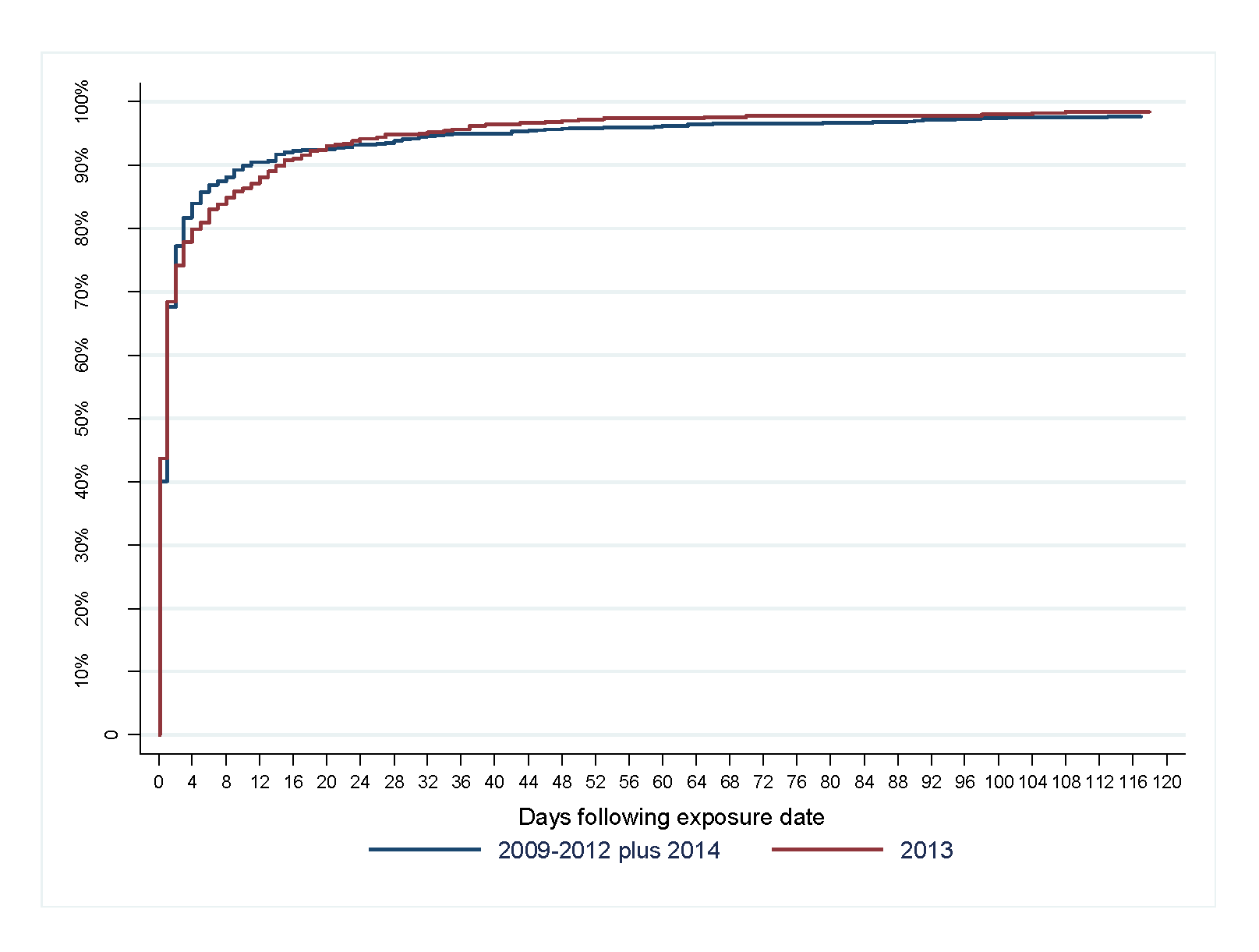

Supplement: S2 Fig — (TIF) [file pntd.0005227.s002.tif]
