# Supplementary material for: Potential Exposures to Australian Bat Lyssavirus Notified in Queensland, Australia, 2009−2014
Source: PLoS Negl Trop Dis. 2016 Dec 29;10(12):e0005227. doi: 10.1371/journal.pntd.0005227 (PMC5199083; doi:10.1371/journal.pntd.0005227)
Supplement: S1 Table — (DOCX) [file pntd.0005227.s003.docx]

| **Hospital and Health Service of residence** | **Number of potential exposures** | **Notification rate***  **(95% confidence interval)** |
| --- | --- | --- |
| Torres and Cape | 16 | 10.8 (6.2–17.5) |
| North West | 12 | 6.3 (3.2–10.9) |
| Cairns and Hinterland | 184 | 12.8 (11.0–14.8) |
| Townsville | 140 | 10.1 (8.5–11.9) |
| Mackay | 64 | 6.1 (4.7–7.8) |
| Central West | 4 | 5.4 (1.5–13.8) |
| Central Queensland | 77 | 6.0 (4.7–7.5) |
| Wide Bay | 68 | 5.5 (4.5–6.9) |
| South West | 9 | 5.7 (2.6–10.8) |
| Darling Downs | 77 | 4.8 (3.8–6.0) |
| West Moreton | 94 | 6.3 (5.1–7.8) |
| Sunshine Coast | 168 | 7.6 (6.5–8.8) |
| Metro North | 221 | 4.1 (3.6–4.7) |
| Metro South | 230 | 3.7 (3.2–4.2) |
| Gold Coast | 151 | 4.7 (4.0–5.5) |
| **Total** | **1,515** | **5.6 (5.3–5.9)** |
